# A cloud-based bioinformatic analytic infrastructure and Data Management Core for the Expanded Program on Immunization Consortium

**DOI:** 10.1017/cts.2020.546

**Published:** 2020-09-22

**Authors:** Sofia M. Vignolo, Joann Diray-Arce, Kerry McEnaney, Shun Rao, Casey P. Shannon, Olubukola T. Idoko, Fatoumata Cole, Alansana Darboe, Fatoumatta Cessay, Rym Ben-Othman, Scott J. Tebbutt, Beate Kampmann, Ofer Levy, Al Ozonoff

**Affiliations:** 1 Precision Vaccines Program, Boston Children’s Hospital, Boston, MA, USA; 2 Division of Infectious Diseases, Department of Pediatrics, Boston Children’s Hospital, Boston, MA, USA; 3 Department of Pediatrics, Harvard Medical School, Boston, MA, USA; 4 PROOF Centre of Excellence, Vancouver, BC, Canada; 5 Vaccines & Immunity Theme, Medical Research Council Unit, The Gambia at the London School of Hygiene and Tropical Medicine, Atlantic Boulevard, Banjul, The Gambia; 6 Vaccine Centre, Faculty of Infectious and Tropical Diseases, London School of Hygiene and Tropical Medicine, London, UK; 7 Telethon Kids Institute, Subiaco, Australia; 8 Centre for Heart Lung Innovation, St Paul’s Hospital, University of British Columbia, Vancouver, BC, Canada; 9 Division of Respiratory Medicine, Department of Medicine, University of British Columbia, Vancouver, BC, Canada; 10 Broad Institute of Harvard and MIT, Cambridge, MA, USA

**Keywords:** Bioinformatics, cloud computing, data management, systems biology, vaccinology

## Abstract

The Expanded Program for Immunization Consortium – Human Immunology Project Consortium study aims to employ systems biology to identify and characterize vaccine-induced biomarkers that predict immunogenicity in newborns. Key to this effort is the establishment of the Data Management Core (DMC) to provide reliable data and bioinformatic infrastructure for centralized curation, storage, and analysis of multiple de-identified “omic” datasets. The DMC established a cloud-based architecture using Amazon Web Services to track, store, and share data according to National Institutes of Health standards. The DMC tracks biological samples during collection, shipping, and processing while capturing sample metadata and associated clinical data. Multi-omic datasets are stored in access-controlled Amazon Simple Storage Service (S3) for data security and file version control. All data undergo quality control processes at the generating site followed by DMC validation for quality assurance. The DMC maintains a controlled computing environment for data analysis and integration. Upon publication, the DMC deposits finalized datasets to public repositories. The DMC architecture provides resources and scientific expertise to accelerate translational discovery. Robust operations allow rapid sharing of results across the project team. Maintenance of data quality standards and public data deposition will further benefit the scientific community.

## Introduction

As scientific technology advances and biomedical research emphasizes big data generation and analysis, an increasing demand for powerful computing capabilities is expected [1]. Such demands may be addressed by purchasing, supporting, and maintaining hardware locally, or more traditionally by dedicated data centers which are costly to establish [2, 3]. There is an unmet need for mid- to large-scale research programs that require customized data management solutions but cannot afford a dedicated data center. Cloud computing has emerged as an appealing approach because of its ease of maintenance, scalability, and on-demand characteristics [3]. Cloud computing is a cost-effective alternative compared to physical hardware-based computing [2]. Multiple backup systems ensure durability and reliability of data with scalability according to demand [4]. Enhanced data security can be implemented internally using application-level best practices while cloud providers enforce external policies [4]. With Findable, Accessible, Interoperable, Reusable (FAIR) guidelines in mind [5], cloud-computing infrastructure can offer retrievable identifiers using standardized protocols with appropriate authentication procedures and ease in sharing data for scientific reproducibility. Efforts and infrastructure to promote FAIR guidelines may in turn help address the perceived scientific crisis of reproducible results which receives frequent comment [6]. As the scope, scale, and complexity of research data increases, integration of computationally intensive data management and biomedical research is likely [7]. The use of cloud computing plays a key role in addressing issues related to traditional storage and analysis of high-dimensional systems biology data [8].

Mid- to large-scale human biomedical studies, for example, those with participants and samples in the hundreds to thousands require a robust data infrastructure to track biological samples along the experimental pipeline, curate and analyze the resulting data files, and share data and results across an inter-disciplinary project team [9]. The *Precision Vaccines Program* Data Management Core (DMC), based at Boston Children’s Hospital, identified these needs while planning the digital infrastructure to support the Expanded Program for Immunization Consortium (EPIC). EPIC is an international affiliation of biomedical centers partnering with the aim of applying systems biology techniques using global molecular tools to identify biomarkers that predict host response to vaccination and/or mechanistic cause–effect of commonly accepted correlates of protection. The initial pilot cohort, designated EPIC-001, demonstrated feasibility of a “small sample – big data” approach using small volumes of human newborn peripheral blood for onsite fractionation and cryopreservation prior to shipment to end point assay laboratories [10]. EPIC received additional funding from the Human Immunology Project Consortium (HIPC), a program established by the National Institutes of Health (NIH)/National Institutes of Allergy and Infectious Diseases (NIAID), to enroll a larger cohort designated EPIC-002 designed to characterize *in vivo, in vitro,* and *in silico* molecular signatures that predict immunogenicity of hepatitis B vaccine in early life.

**Fig. 1.**
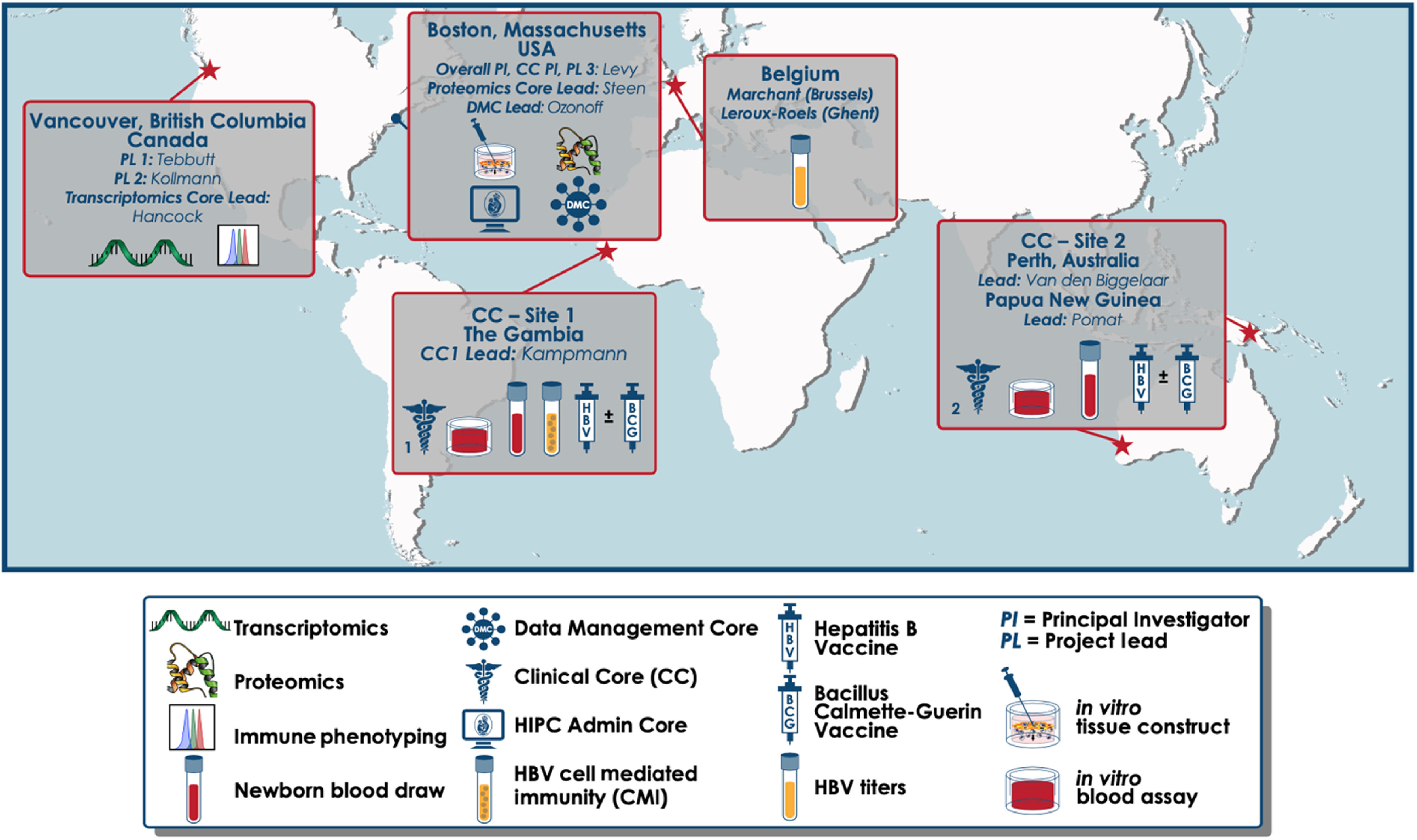
Global map of our Administrative, Clinical, Service, and Data Management Cores.

The EPIC-HIPC project was organized with multiple cores around the world working collaboratively to fulfill our specific aims (Fig. 1), including an Administrative Core, a Clinical Core, and several Service Cores to perform experimental assays including a Proteomics Core (Boston, MA) and Transcriptomics Core (Vancouver, BC). The DMC established three scientific aims for this project: (1) create a project-wide secure data management infrastructure; (2) provide a cloud-based scientific environment to enable cross-platform bioinformatics and integrative analyses; and (3) establish EPIC-HIPC-wide quality assurance (QA) policies and standards for each data source. The DMC’s core functional responsibilities included accurate and reliable data capture, secure data management, QA, project and analytic computing resources, and deposition of data to public repositories.

Amazon Web Services (AWS) offers an on-demand, scalable, and secure cloud-computing platform which includes several services to meet our project needs. For data storage, we used Amazon Simple Storage Service (S3) which is an object storage service offering scalability and continuous data availability [11]. We used AWS Elastic Compute Cloud (EC2), a service that provides secure and web-scalable cloud computing, to host our software platforms, e.g., sample tracking software and analytic computing environment [12]. Using AWS Identity and Access Management (IAM), we managed user permissions and access and restricted the inbound security groups with access to our EC2 instances [13].

## Materials and Methods

We specified three core principles to guide DMC implementation for the infrastructure design: data security, operational flexibility, and affordability. Our design, based on these three main themes, maintains a robust data platform that addresses unique project needs.

### Data Security

Data security and privacy are critical concerns when managing data from human study participants. In partnership with AWS, our institution established a Business Associate Agreement (BAA) allowing a regulated and secure AWS environment to process, maintain, and store protected health information, as required under U.S. Health Insurance Portability and Accountability Act of 1996 rules [14]. Through AWS security features, we restricted access to EPIC collaborators only using a controlled list of Internet Protocol (IP) addresses. We further required user credentials specific for each research team member for each service used. For data storage, the DMC architecture leveraged S3 as a local data repository. For data integrity and version control, we designated permissions such that only DMC administrators had download or deletion privileges. All other project users had ‘push’ privileges only, i.e., users could upload but not download or delete files. We installed a number of server-based software products maintained on EC2 virtual instances. This design allowed us to maintain multiple software and applications, store and share data securely, and scale or remove instances as project computing needs changed. Each EC2 instance or S3 bucket was assigned a security group which acted as a virtual firewall to control all communications via a specified list of inbound rules [15]. Secured access and central repository of all datasets were managed by DMC staff. Data were secured through encryption, controlled IP access, and user credentials.

### Operational Flexibility and Low Cost

We intended our model to be adaptable and scalable to unforeseen needs. Cloud computing offers a flexible approach to infrastructure design to enable payment for services on-demand and to scale.

**Fig. 2.**
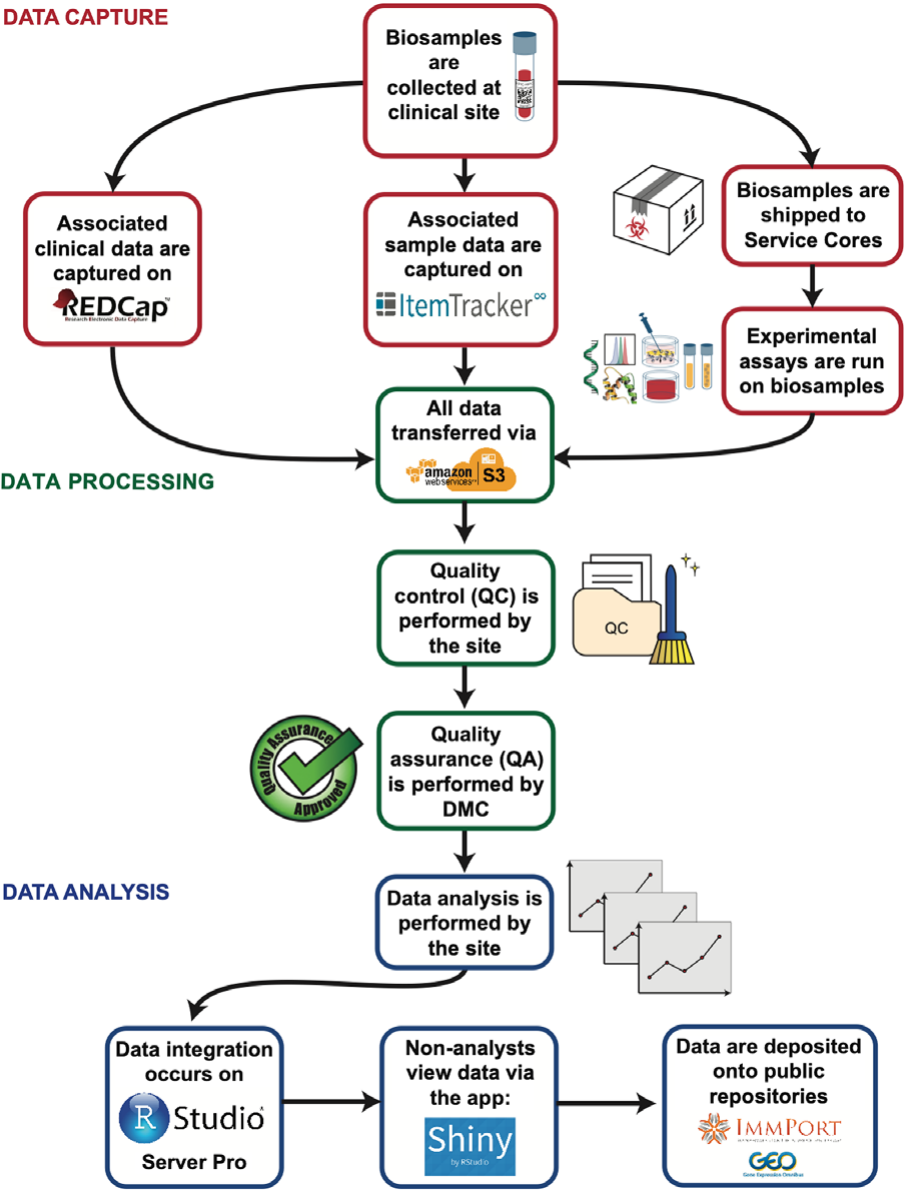
Overall data pipeline for the project. Clinical and sample data are generated and captured at the clinical site. Experimental assays are run in multiple Service Cores. Each of these sites and cores performs quality control (QC) as well as independent data analysis. All data transfers occur via S3. The DMC then performs quality assurance (QA) and uploads the clean data to S3. Data are integrated on RStudio Server Pro and accessed on R Shiny application. Following publication of study output, data are deposited onto public repositories, such as ImmPort and Gene Expression Omnibus. Note: This illustration does not necessarily depict chronological timelines as the data flow is often run in multiple batches.

We designed the digital infrastructure to support EPIC-HIPC studies around three broad categories of activity: data capture, data processing, and data analysis (Fig. 2). We considered options for computing platforms and software to achieve our design aims and ultimately selected a system built upon AWS cloud-computing architecture. This allowed us to self-service implementation and maintenance of our system, using the AWS web-based graphical interface for system configuration and administration. Integration of data storage with computing facilities was a useful feature of AWS architecture. Cost estimates suggested that AWS would provide a cost-effective solution relative to other options.

The main alternative to cloud computing that we considered was local server hosting; yet, we found this option to be less efficient and more costly. A local server requires purchase and replacement of hardware, skilled staff to configure and maintain the system, and dedicated laboratory space to house hardware. Moreover, such an approach would require an upfront investment and routine maintenance throughout the project period, whereas the pay-as-you-go approach of cloud computing incurred low costs early in the project period during enrollment and sample tracking. Because of the previously established BAA with our institution, AWS was a natural choice of platform to avoid the time and expense and to establish the necessary agreements with another cloud-computing service management company, e.g., Microsoft Azure [16] or Google Cloud [17]. Our institution has a Research Computing team with extensive AWS experience that provided support and advice during the design and deployment of our digital infrastructure.

### Data Capture

The clinical information and sample metadata were captured at the clinical sites using electronic case report forms (eCRFs) and sample processing forms (SPF), respectively. Data captured on the eCRFs are described in our clinical protocol [18]. Metadata captured on the SPF include basic sample metadata such as the date/time of collection, study personnel involved in sample collection, and a unique identifier used to link biosamples to clinical data.

Traditionally, clinical data were captured on paper case report forms (CRFs); however, improvised eCRFs are now preferred [19]. Within the scope of our project, we captured clinical data in a custom-built Research Electronic Data Capture (REDCap) database [20, 21] designed and developed in collaboration with the Clinical Core. REDCap is a secure, web-based software platform to support data capture for research studies, providing (1) an intuitive interface for validated data capture; (2) audit trails for tracking data manipulation and export procedures; (3) automated export procedures for seamless data downloads to common statistical packages; and (4) procedures for data integration and interoperability with external sources [20, 21]. Although REDCap has the technical means to provide compliance with FDA 21 CFR Part 11, we did not implement those features in this study [21, 22]. There are many alternatives to REDCap, e.g., Studytrax [23] and InForm Electronic Data Capture [24]. Our decision relied on the academic availability and current implementation across our institution and the clinical sites at The Gambia and Papua New Guinea.

After collecting biological samples at the clinical sites, we tracked each sample point-to-point with commercial software *ItemTracker* [25] implemented via user-accessible Windows remote desktop or web-based application, both of which store data on a Microsoft SQL database hosted on an EC2 instance. We updated *ItemTracker* with the sample infrastructure defining each entry using preloaded numerical item identifiers for participants. We uploaded sample label sets, consisting of predefined study visit sets, into a project-specific *ItemTracker* configuration. Each study visit set was uniquely identified using a randomized four-digit alpha-numeric visit identifier. All samples were assigned a unique item identifier at the time of *ItemTracker* upload. We provided clinical sites with unassigned sample label sets for sample collection. Preprinted labels included information on the type of sample, the unique alpha-numeric visit identifier, and a scannable Quick Response (QR) code with embedded identifier data. We captured sample metadata (e.g., time of collection, plasma volume, laboratory technician initials) and linkage between the sample and subject identifier on paper SPFs which we entered manually into *ItemTracker*.

The DMC used *ItemTracker* to track all biological specimens as they were shipped from clinical sites to Service Cores. Sample locations were updated by scanning a QR code on a box of samples or the sample tube itself. As boxes of samples were shipped, they were placed in an “In Transit” folder which was then updated upon delivery and receipt. The samples were initially stored in the order they were collected, but once they reached the Service Cores, they were sorted chronologically following a sorting map generated by the DMC.

**Fig. 3.**
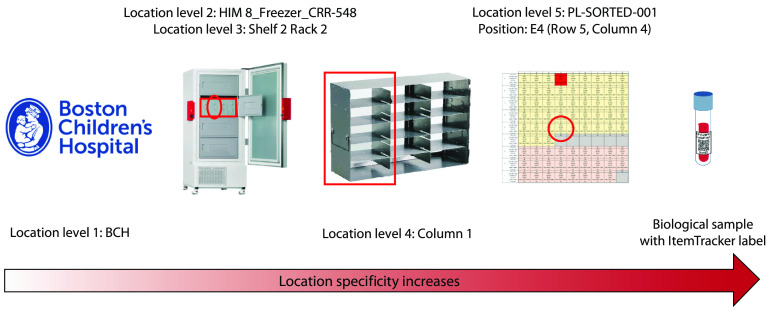
The multi-level location hierarchy established in ItemTracker. The example above illustrates a biological sample with its location coded as: BCH/HIM 8_Freezer_CRR-548/Shelf 2 Rack 2/Column 1/PL-SORTED-001/5/4/E4.

Sample locations were tracked using a multi-level hierarchy including site, building, room, storage freezer, storage shelf, storage rack, storage column, box number, and position within box (Fig. 3). Once the samples reached their final destination and were sorted, Service Cores ran each sample through experimental assay pipelines. EPIC-HIPC collaborators conducted multiple assays including transcriptomics, proteomics, flow cytometry, and antibody titers within dedicated Service Cores (Fig. 1).

### Data Processing

Effective data management is essential to make data discoverable, accessible, and understandable [26]. Each Service Core specified and performed data curation according to each data type and generated initial quality flags for data analysis. To clearly define our terminology, quality control (QC) refers to the local processes to establish data quality standards performed at the Service Cores, while QA is the central process to verify and ensure data quality by the DMC.

Once the data completed local QC, core and site researchers uploaded data to the S3 directory, including associated “readme” text files to explain the format and contents of each data file. There were data validation checks embedded into the REDCap data capture system to identify and flag missing or out-of-range values. The DMC performed additional QA processes to all clinical, sample, and experimental data. QA was specific to each data type, and generally included quality checks across the following themes: verifying QC processes, checks for missing values, identification and investigation of outliers, chronologic deviations, i.e., date and time measures, and protocol deviations. Through the QA process, the DMC generated relevant flags for data analysts and then uploaded the final datasets to S3.

As QA was completed, data are deposited in real time to public repositories (e.g., ImmPort [27] and Gene Expression Omnibus [28]), set for public release upon publication. For example, the data from EPIC-001 are available at ImmPort (immport.org) under study accession SDY1256 and SDY1412 [10, 27]. The DMC established consistent file naming conventions for each data type to ensure standardization and reproducibility, setting guidelines of good practice and facilitating the deposition of data to public repositories in order to maximize benefit to the broader scientific community.

### Data Analysis

The DMC supports project cores and scientists responsible for data analysis. All project analysts used RStudio Server Pro hosted on an EC2 instance [29]. This analytic platform seamlessly integrated data stored on S3 for a controlled repository and cloud-computing environment that ensured repeatable and reproducible results.

Data visualization is crucial to convey results and information, yet not all project scientists had the computing experience to conduct analyses directly from raw data. RShiny, a user-friendly application, allows scientists to visualize data interactively from a centralized platform [30]. We included a variety of widgets to empower the users to control their visual outputs, e.g., radio buttons or drop-down menus to select from a list of analytic options. Using these widgets, the users specified graphical outputs such as color graphs of specified data sources. We further added a feature to allow users to hover over a point of interest in a graph and return a table providing the data associated to said point. The RShiny application was hosted on an EC2 instance with a security group containing specified inbound rules.

## Results

We implemented *ItemTracker* to track over 45,000 tubes containing human samples collected and shipped internationally for our cohort in The Gambia (*n* = 720). Initially, we hosted the software on a Windows m4.large instance (two virtual central processing units (vCPUs) and eight GiB memory). Once we reached computing capacity, noted by lag and latency experienced by simultaneous users, we upgraded the instance to m4.xlarge (4 vCPU and 16 GiB memory). We uploaded all study data to designated S3 directories that linked reliably with the RStudio Server Pro platform hosted on an EC2 instance. Overall storage accounted for over 100,000 data files with an estimated two terabytes of storage. Similarly, our computing instance that hosted RStudio Server Pro initially used a Linux m4.large instance (2 vCPUs and 8 GiB memory). As our computing needs expanded, we upgraded to m5.xlarge (4 vCPUs and 16 GiB memory). We added capabilities to provide additional short-term resources for time-limited high-intensity computing. For example, a single run of a biomarker discovery pipeline might require dozens of processing cores and an order of magnitude increase in memory allocation over a period of 1–2 weeks. By allowing time-flexible scalability in both directions, we avoided costly investments in server architecture, while maintaining local control of a dedicated environment rather than relying on a shared resource such as a high-performance cluster. To further reduce cost, we scheduled EC2 instances to deactivate outside of typical working hours.

To prioritize our QA activities, we categorized the clinical data into four categories:

- DMC-internal clinical data are used for variable derivation and/or QA purposes – e.g., date and time stamps or inclusion and exclusion criteria.
- Tier 1 clinical data are critical to answer proposed primary study questions – e.g., randomization group assignment or biological sex.
- Tier 2 clinical data are question-specific data – e.g., breastfeeding status.
- Tier 3 clinical data are exploratory data – e.g., physical assessment of neonate.

Throughout our comprehensive QA processes, we generated multiple queries to ensure data quality available to researchers. For example, for the Tier 1 and DMC-internal clinical data encompassing 177 variables (columns) for 720 subjects (rows), we generated eight data quality reports over 9 months, containing 149 queries, and 82 of these queries (55%) led to data changes. The timeline for data to complete QA was dependent on various factors, e.g., when the data files were received, the QC process of the associated Service Core, the quality level of the resulting data, the size of the data, the QA processes performed on a specific type of data, the response time to QA queries, and the overall bandwidth of the DMC as we processed multiple datasets simultaneously. Conducting diligent QC/QA processes according to a standard protocol maintained high data quality while creating notable pressure to meet expected timelines. The DMC worked efficiently to balance timelines with data quality. For example, we defined tiers of clinical data to prioritize QA and expedite data availability such that the most important subsets of clinical data moved through our QA process immediately while ancillary variables were deferred. Similarly, we established a high priority to complete QA for experimental assay data as it became available to enable ongoing data analysis.

**Fig. 4.**
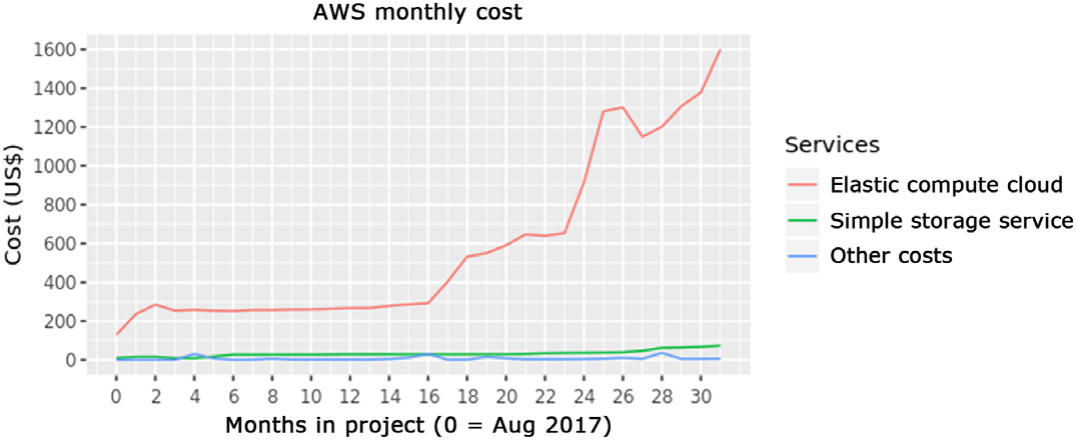
Cloud computing costs over the first 31 months of the EPIC-HIPC project. The starting date is August 2017.

We tracked monthly costs associated with the study’s data infrastructure throughout the course of the project (Fig. 4). During the period of clinical cohort enrollment and sample tracking, monthly costs remained below $300 USD. There were additional fixed costs for the sample tracking database below $5k USD per year. As the project team engaged in more data analysis, monthly costs increased. The DMC conducted development and user testing of the shared analytic resources during project months 16 to 23. Starting in project month 24, analytic usage increase and monthly costs grew accordingly. During these periods, there were additional fixed annual costs for computing software licenses below $10k USD per year.

## Discussion

The DMC established and maintained a cloud-based discovery environment, consisting of data storage and computational tools to perform integrative systems analyses and facilitate collaborations between the projects and cores. Dealing with design complexity and implementation of this infrastructure, we experienced several challenges.

Tracking of biological samples using *ItemTracker* seemed straightforward; however, when faced with real-world conditions, we saw inevitable complications. Due to the nature of the sample collection, processing, and storage, timeliness of processing affected sample integrity. We updated the sample processing protocol to allow faster sample storage at the clinical sites. The status of samples was updated manually after experimental assays were processed, which made it difficult to maintain accurate status updates in real time. DMC staff addressed this challenge with frequent communication to cores and subsequent data queries during QA once it was clear that sample locations were out of date. We delivered additional training to sites on study processes and the use of the tracking platform to ensure all updates were performed according to protocol. Non-project research staff at some sites occasionally relocated sample boxes, leading to further location data inaccuracies. We addressed this issue with a dedicated freezer area for the project at sites where this was feasible. Overall, the DMC identified operational challenges and partnered with the core sites to solve or mitigate these issues.

Implementation of RStudio as the primary software platform to analyze project data posed a collaborative challenge. While most data analysts were comfortable with the platform, some of the project-affiliated biomedical researchers were not familiar with the R programming language. The DMC developed an RShiny application with pre-generated graphical outputs to allow for data exploration and visualization. The application’s interactive features allowed researchers without coding experience to perform predefined analyses and visualizations – e.g., plotting data by biological sex. The DMC intends to develop and integrate other software platforms to broaden the usability of the architecture outside of those analysts familiar with R.

When providing computing support for the smaller pilot cohort (EPIC-001, n = 30, 2 timepoints) [10], we used a decentralized model that allowed analysts the convenience of directly accessible data. This decentralization resulted in naming inconsistencies and versioning conflicts across multiple instances of data files. This posed challenges to the QA process, verification of analyses for purposes of reproducibility, and the eventual process of data deposition. The loss of central control over data files had further implications for data security.

Throughout the conception and design of the data architecture for our main study cohort (n = 720), the DMC maintained data governance focusing on a centralized model for data access and management. Although data security was a prominent feature in our design, there were consequent trade-offs with data accessibility. The flexibility of our infrastructure allowed us to balance these competing principles. We set a clear framework for implementation and communicated our core principles to the project team while responding to feedback from users.

*ItemTracker* deployed on AWS platform proved to be a robust and reliable software platform as demonstrated by the large quantity of biological samples tracked. We implemented a hybrid approach for QC/QA which decentralized QC and centralized QA. We relied on the scientific expertise of each Service Core to perform QC locally and provide data of high quality to the DMC. Each core offered recommendations for additional QA by the DMC and feedback to improve the process. This additional layer of QA improved the overall quality of the data and analytic pipeline, as evident by the number of queries submitted and eventual data edits.

A notable limitation of our design was the lack of consistent metadata capture related to DMC operations. Although we collected limited data on key performance measures as reported above, we did not have reliable capture of personnel time spent on specific processes, nor did we establish a systematic approach to classify QA queries and their resolutions. We have encountered some resistance to strict adoption of our infrastructure and guidelines for use, as is often the case with large collaborative scientific projects. Implementation challenges demonstrated the complexity of our application. We believe our design offered flexibility and balanced usability while staying faithful to our core principles.

Our experience suggests that cloud computing is a suitable approach for mid-scale collaborative projects with modest financial budgets. Scientific endeavors of this scope/scale require robust data management plans, infrastructure, and operations. Implementing centralized data governance with selected decentralized operations proved a feasible and flexible approach that provided both data security and accessibility. We believe our approach offers advantages over a more traditional server-based architecture, most notably an efficient and effective computing environment for integrative analyses and scientific discovery.
